# Clinical Evaluation of Resin Composite and Resin Modified Glass Ionomer in Class III Restorations of Primary Maxillary Incisors: A Comparative *In Vivo* Study

**DOI:** 10.5005/jp-journals-10005-1024

**Published:** 2009-08-26

**Authors:** Usha Mohan Das, Deepak Viswanath, Umme Azher

**Affiliations:** 1 Principal, Professor and Head, Department of Pedodontics and Preventive Dentistry, VS Dental College and Hospital KR Road, VV Puram, Bengaluru-560004, Karnataka, India; 2 Assistant Professor, Department of Pedodontics and Preventive Dentistry, VS Dental College and Hospital, KR Road VV Puram, Bengaluru-560004, Karnataka, India; 3 Senior Lecturer, Department of Pedodontics and Preventive Dentistry, VS Dental College and Hospital, KR Road, VV Puram Bengaluru-560004, Karnataka, India

**Keywords:** Resin-modified glass ionomer cement, composite resin.

## Abstract

Restoration of primary teeth continues to be an important facet of restorative dentistry. In comparison to restorations in permanent dentition, the longevity of those in primary teeth is significantly different for all materials. This makes the assessment of these fillings as a separate group meaningful. As there is lack of supporting clinical data with regard to the restoration of primary incisors, it would be judicious to consider why this is so and determine if studies can be designed to gain new information. The purpose of this study was therefore to evaluate and compare the clinical efficacy of composite resins and resin-modified glass ionomer cement restorations of primary incisors, over a period of one year.

*Methods:* The study group consisted of 40 patients (3½- 5 ½ years of age) with at least one pair of similar sized lesions in the middle1/3 of the same proximal surface of contralateral primary maxillary incisors. Composite resin and resinmodified glass ionomer cement restorations were placed in primary maxillary incisors using split-mouth design. The restorations were evaluated at different intervals of 3,6,9, months and 1 year using Ryge’s criteria. Data obtained was analyzed using Mann-Whitney test.

*Results:* The results revealed no statistical significance in the difference of clinical characteristics between the two restorative materials.

*Interpretation and conclusion:* (1) Resin-modified glass ionomer cement and composite resin restorative materials showed acceptable clinical performance after 1 year in primary teeth. (2) Resin-modified glass ionomer cement and composite resin restorative materials functioned well as class III restorative materials in primary teeth.

## INTRODUCTION

The practice of dentistry for children is an integral component of children’s health care. Generally dentists and pediatric dentists have been providing this type of care with the intent to provide optimal oral health for children. Although the dental profession has been successful in decreasing the amount of dental disease in children with the aid of community water fluoridation and increased public awareness of dental disease prevention, a recent surgeon’s general report on oral health said that there is still is a tremendous ongoing need for pediatric dental care.^1^

Restoration of primary teeth continues to be an important facet of restorative dentistry. In comparison to restorations in permanent dentition, the longevity of those in primary teeth is significantly different for all materials. This makes the assessment of these fillings as a separate group meaningful.

In general it is the unique morphology of the primary incisors that is the principal deterrent in their restoration with any dental material available today including the latest in composite resins. When applied to the primary incisors, composite resins can be misused and abused unless there is careful case selection, cavity preparation, and material placement.

In a literature review presented by Lee, there is very little long-term, controlled clinical data which validates or endorses any of the restorative options for repairing carious primary anterior teeth. Operator preferences, esthetic demands by parents, the child’s behavior, and moisture control are all variables which affect the decision and ultimate outcome of whatever restorative treatment is chosen.

## AIMS AND OBJECTIVES

1. To evaluate clinical efficacy of composite resin class III restorations in primary incisors.
2. To evaluate clinical efficacy of resin-modified GIC class III restorations in primary incisors.
3. To compare clinical efficacy of resin-modified GIC vs composite resins in primary incisors.

## MATERIAL AND METHODS

The present study was a nonrandomized comparative study conducted in the Department of Pedodontics and Preventive Dentistry, VS Dental College and Hospital, Bengaluru, India. 40 patients in the age group of 3½-5½ years age who were dependable on recall appointments with at least one pair of similar sized lesions on the middle-third of the same proximal surface of contralateral primary maxillary incisors were selected.

Exclusion criteria:

1. Teeth with deep caries lesion.
2. Presence of soft tissue abscess or sinus tract around the teeth.
3. Teeth which are not restorable.
4. Patients with anterior teeth malocclusions.
5. Patients with oral habits.
6. Mentally challenged patients.

The samples were divided into 2 groups:

*Group 1:* Forty teeth to be restored with GC Fuji FillingTM LC in class III cavity preparation in primary maxillary incisors.

*Group 2:* Forty teeth to be restored with SOLARE in class III cavity preparation in primary maxillary incisors.

## METHODOLOGY

### Cavity Preparation

**Fig. 1:**
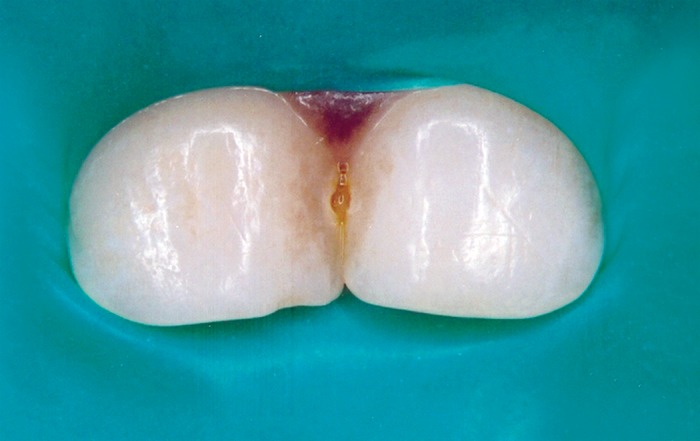
Isolation of teeth under rubber dam

**Fig. 2:**
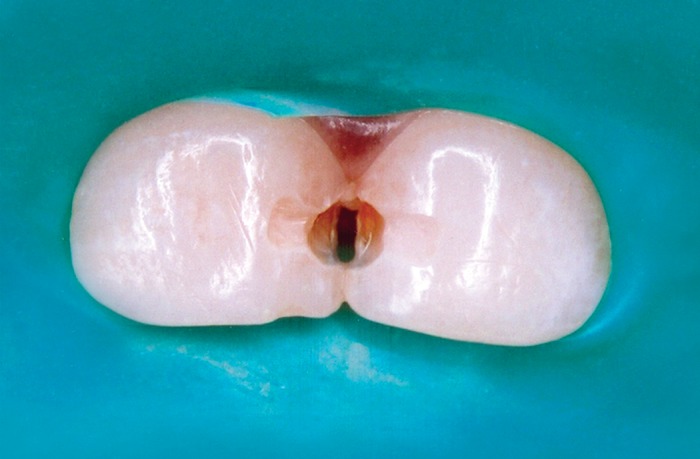
Class III cavity preparation irt 51 and 61

1. The child’s attention was diverted using compact disk player, personal earphones, and a compact disk of children’s songs. Using a resin-based composite shade guide, a suitable color-filled resin was selected.
2. The teeth were isolated using rubber dam (Fig. 1).
3. A wooden wedge was positioned to retract the proximal dam material and protect underlying gingival tissues. during tooth preparation. Access to the lesion was made from the labial aspect. Debridement of carious substance was completed using a slow-speed round bur, and the outline form was cut using a water-cooled, straight fissure diamond bur at high speed. Outline form included a small labial dovetail preparation to add mechanical interlocking retention form to the cavity design (Fig. 2). In the incisogingival direction the preparation is extended to include the defect and place the walls of the cavity preparation in sound enamel and dentin.
4. Peripheral enamel was roughened with a slow-speed tapered diamond bur.

### Procedural Steps for Composite Resins

1. Tooth was polished with pumice slurry.
2. After polishing a celluloid matrix band was placed on the proximal surface of the tooth.
3. A small applicator tip was used to rub self-etching primer solution within the preparation and peripheral surfaces for 20 seconds.
4. Bonding agent was applied using applicator tip and cured using light beam for 20 seconds.
5. Composite resin (SOLARE) was placed in increments and cured for 40 seconds.
6. Restoration was finished and polished.

### Procedural Steps for Resin-Modified GIC

1. Tooth was polished with pumice slurry.
2. After polishing a celluloid matrix band was placed on the proximal surface of the tooth.
3. The cavity was conditioned using a non-rinse GC cavity conditioner for 10 seconds.
4. Resin-modified GIC (Fuji Filling TM LC) was placed and cured for 40 seconds.
5. Restoration was finished and polished.

**Fig. 3:**
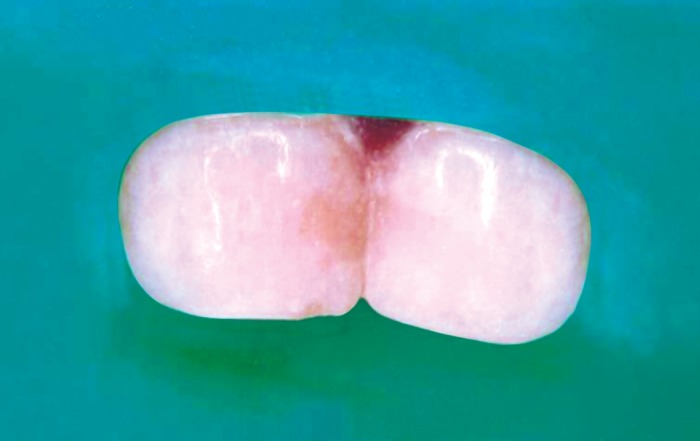
Restoration of 51 with RMGIC and 61 with composite resin

The patients who were treated with both composite resin (SOLARE) and resin-modified GIC (Fuji Filling TM LC) (Fig. 3) were evaluated at 3,6,9 and 12 months using Ryge’s criteria (Table 1).

## STATISTICAL ANALYSIS AND RESULTS

Mann-Whitney test was carried out to find out if there was any significant difference between the scores, obtained for the two restorations at different time intervals. And to compare the scores of RMGIC and composite resin at different time intervals under each parameter - Wilcoxon Signed-Rank test.

**Graph 1:**
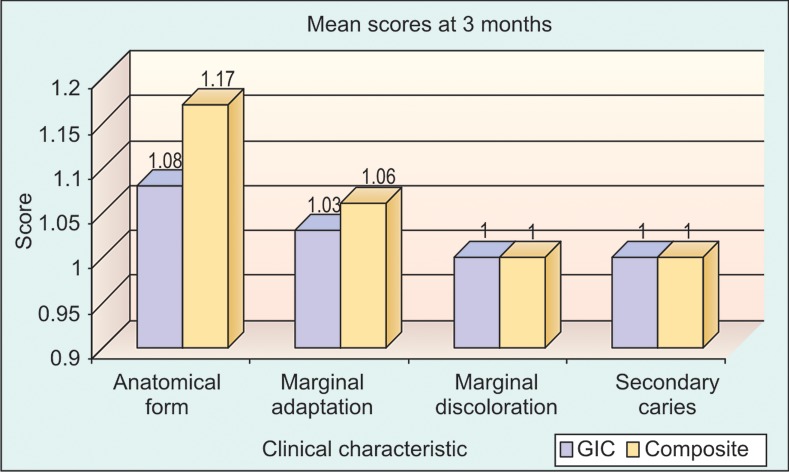
There was no significant difference between RMGIC and composite with respect to anatomical form (p > 0.05), marginal adaptation (p > 0.05), marginal discoloration (p > 0.05) and secondary caries (p > 0.05) at 3 months time interval

**Graph 2:**
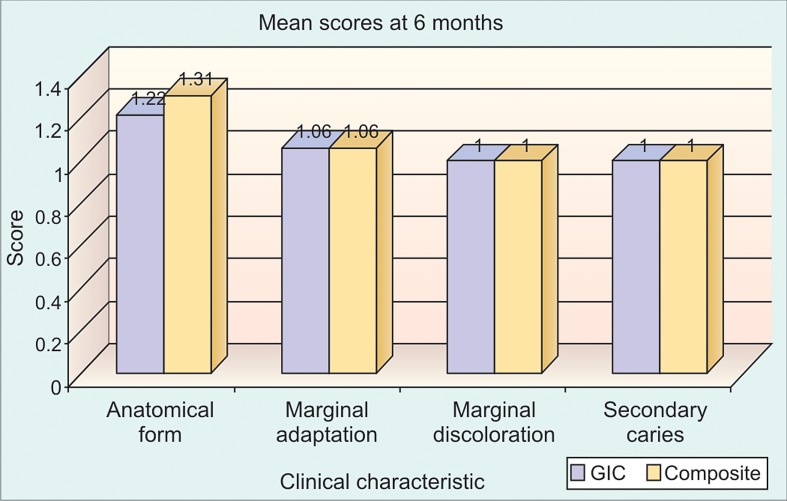
No significant difference between RMGIC and composite with respect to anatomical form (P > 0.05), marginal adaptation (p > 0.05), marginal discoloration (P > 0.05) and secondary caries (p > 0.05) at 6 months time interval

**Table 1:** Table 1: Rating system and criteria for evaluating of clinical characteristics of the restorations (Ryge-1980)

*Clinical characteristics*	*Category of rating*	*Evaluation criteria*
Marginal adaptation	A	No catch or visible evidence of a cervice along the margin A small catch, crevice or ditch but dentin or cement base is not exposed
		A small catch, crevice or ditch but dentin or cement base is not exposed
	B	Dentin or cement base is exposed
		Mobile restoration, fractured or missing in part or total
	C	
	D	
Anatomic form	A	Restoration contour is continuous with existing anatomic form
	B	Restoration is under contoured, restorative material discontinuous with the existing anatomic form but loss of material not sufficient to expose dentin or base
	C	Loss of material to the extent that dentin or base is exposed
Secondary caries	A	No evidence of caries contiguous with the margin of the restoration
	B	
Margin discoloration	A	No discoloration penetrated along the margin of the material in a pulpal direction
	B	Discoloration penetrated along the margin of the material in a pulpal direction
The restorations were evaluated for anatomical form, marginal adaptation, marginal discoloration and secondary caries at different time intervals of 3 months, 6 months, 9 months and 1 year.		

**Graph 3:**
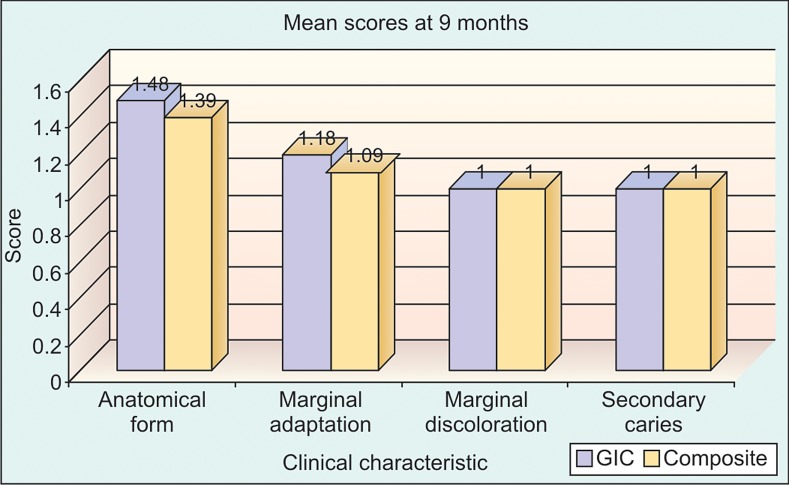
No significant difference between RMGIC and composite with respect to anatomical form (P > 0.05), marginal adaptation (P > 0.05), marginal discoloration (P > 0.05) and secondary caries (P > 0.05) at 9 months time interval

## DISCUSSION

One of the important aims in restorative dentistry is to conserve tooth structure during the cavity preparation and removal of caries. Restoration of primary teeth continues to be an important facet of restorative dentistry. Class III restorations of primary incisors can be quite challenging; due to the small clinical crown, the relatively large size of the pulp chamber, the close proximity of the pulp horns to the interproximal surfaces, and the thinness of the enamel, repairing interproximal decay in these teeth require preparations that are conservative in depth with close attention to detail, both to the preparation itself and to the material placement.^2^

**Graph 4:**
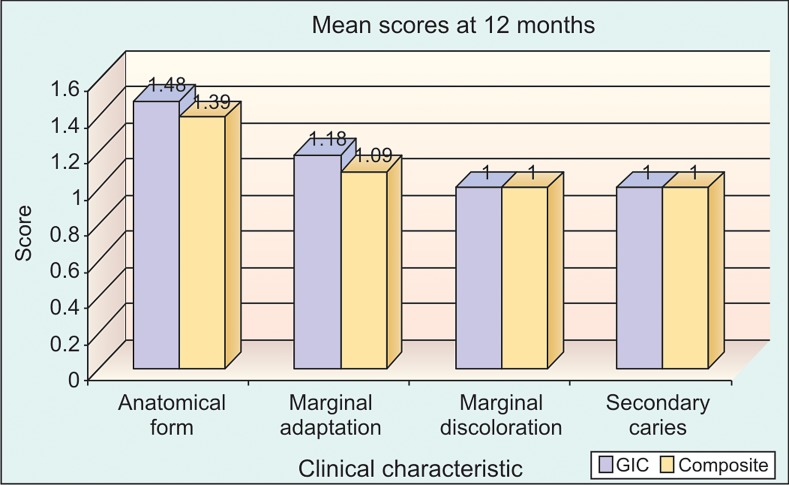
No significant difference between RMGIC and composite with respect to anatomical form (P > 0.05), marginal adaptation (P > 0.05), marginal discoloration (P > 0.05) and secondary caries (P > 0.05) at 12 months time interval

The technique sensitivity of placing class III esthetic restorations is very high. Moisture control, hemorrhage control from the gingival, and retention of the rubber dam are all challenges to be overcome to get successful result. When removing interproximal decay on primary incisors for a class III restoration, keeping a very small conservative preparation-such as slot preparation- may not be the best choice.^3^

The composite resins have found wide acceptance as replacements for the acrylic or unfilled resins and silicate cements for the esthetic restoration of the primary and permanent anterior teeth.^4^-^6^ They offer a host of advantages over acrylic and silicate cements; be it dimensional stability (there is improved marginal adaptation and integrity, with less marginal stain, recurrent caries and lost restorations from the small cavity preparations) or color stability and lastly the composite resins are not only harder but also stronger than the acrylic resins. As a result they are more resistant to wear when placed in areas of occlusal stress. Composite resins are, in fact, the first adequate tooth colored filling material available to restore the anterior teeth. However, there are limitations and extremes to which these materials can be subjected when applied to primary incisors.

Resin-modified glass ionomer cements were developed around 1990s from conventional glass ionomer cement by adding components such as bis-glycidyl methacrylate (bis- GMA), hydroxyethyl methacrylate (HEMA), or a water/ HEMA mixture to replace some of the water in conventional GIC, to improve mechanical properties of GIC, to have better fracture toughness and wear resistance compared to GIC as well as higher moisture resistance and longer working time. The RMGIC restoratives have a continuous fluoride release and thus a potential cariostatic effect, although the longterm fluoride release might be somewhat reduced compared to GIC.

Therefore the present *in vivo* study was performed to assess the clinical evaluation of resin composite and resinmodified glass ionomer cement in class III restorations of primary maxillary incisors. Our study comprised of 40 patients in the age group of 3½ to 5½ years with at least one pair of similar sized lesions on the middle 1/3 of same proximal surface of contralateral primary maxillary incisors. Factors such as age of the patient, co-operation of the parents and behavior of the child patient were all taken into consideration during our study.

In our study we compared the scores from resin-modified glass ionomer cement and composite resin with respect to anatomic form, marginal adaptation, marginal discoloration and secondary caries at different time intervals of 3 months (Graph 1), 6 months (Graph 2), 9 months (Graph 3) and 12 months (Graph 4) using Mann-Whitney test.

## ANATOMIC FORM

The results at the end of 3 months showed for anatomical form, resin-modified glass ionomer cement had success percentage of 91% and for composite resin it was 88% (p = 0.438), when compared to a study done by de Araujo et al,^7^ where they reported a success rate of 100% for both the restorative materials.

At the end of 6 months, resin-modified glass ionomer cement had a success percentage of 82% and composite resin had a success percentage of 80% (p = 0.549), as compared to a study done by Brackett et al,^8^ where they reported a success rate of 94% for resin-modified glass ionomer cement and 100% for composite resin.

At the end of 9 months, resin-modified glass ionomer cement had a success percentage of 65% compared to composite resin which had 74% (p = 0.497). In comparison, a study done by de Araujo et al^7^ reported a success rate of 95% for GIC and 100% for composite resins.

At the end of 1 year, we recorded a success percentage of 65% for resin-modified glass ionomer cement and 74% for composite resin (p = 0.497). A similar study done by Brackett et al^8^ showed a success percentage of 93% for resinmodified glass ionomer cement and 96% for composite resin.

Further a similar study done by de Araujo et al^7^ also reported a success percentage of 90.4% for GIC and 100% for composites.

Our study revealed that there was no significant difference between resin-modified glass ionomer cement and composite resin with respect to anatomical form during all the four evaluations (p > 0.05).

In our study in order to compare the scores of RMGIC and composite resin at different time intervals under each parameter we used Wilcoxon-Signed Rank test.

For anatomic form, we observed that, there was a significant difference between the scores of RMGIC between 3rd and 6th month (p < 0.05), 3rd and 9th month (p < 0.01), 6th and 9th month (p < 0.01), 3rd and 12th month (p < 0.01), 6th and 9th month (p < 0.01) and 6th and 12th month (p < 0.01). However there was no significant difference between 9th and 12th month.

For composites there was a significant difference between the scores of RMGIC between 3rd and 6th month (p < 0.05), 3rd and 9th month (p < 0.05), 6th and 9th month (p < 0.05), 3rd and 12th month (p < 0.05). But there was no significant difference between other time intervals (p < 0.05).

## MARGINAL ADAPTATION

The results at the end of 3 months showed a success percentage of 97% for resin-modified glass ionomer cement and 94% for composite resins (p = 0.558).

Our results showed, at the end of 6 months both resinmodified glass ionomer cement and composite resin had a success percentage of 94% (p = 1.00); as compared to study done by Brackett et al,^8^ where they reported a success rate of 94% for resin-modified glass ionomer cement and 74% for composite resin.

At the end of 9 months, resin-modified glass ionomer cement reported to have a success rate of 88% and composite resin 94% (p = 0.396).

At the end of 1 year our study recorded a success percentage of 88% for resin-modified glass ionomer cement and 94% for composite resin (p = 0.396); compared to study done by Brackett et al^8^ where they recorded a success percentage of 87% and 88% for resin-modified glass ionomer cement and composite resin respectively.

Our study revealed that there was no significant difference between resin-modified glass ionomer cement and composite resin with respect to marginal adaptation during all the four evaluations (p > 0.05).

In our intraindividual comparison of scores for RMGIC at different time intervals for marginal adaptation we observed that there was no significant difference in the scores at different time intervals (p > 0.05). Similar results were obtained from composite resin restorations at different time intervals (p > 0.05).

## MARGINAL DISCOLORATION

The results at the end of 3,6,9 and 12 months showed resinmodified glass ionomer cement and composite resin had a success percentage of 100% (p = 1.00), which are in par with results achieved by de Araujo et al,^7^ where they showed similar results.

Our study revealed that there was no significant difference between resin-modified glass ionomer cement and composite resins with respect to marginal discoloration during all the evaluations (p > 0.05).

In our intraindividual comparison of scores for RMGIC at different time intervals for marginal discoloration we observed that there was no significant difference in the scores at different time intervals (p > 0.05).

Similar results were obtained from composite resin restorations at different time intervals (p > 0.05).

## SECONDARY CARIES

The results at the end of 3,6,9 and 12 months showed resinmodified glass ionomer cement and composite resin had a success percentage of 100% (p = 1.00), which are in par with results achieved by Ozgunaltay et al,^9^ and Kitty MY et al,^10^ who also reported similar results.

Our study revealed that there was no significant difference between resin-modified glass ionomer cement and composite resin with respect to secondary caries (p > 0.05).

The clinical evaluation at 1 year showed that resinmodified glass ionomer cement had significantly decreased marginal integrity compared to composite resin, but the difference was statistically insignificant. Both the materials were rated clinically acceptable for anatomic form, marginal adaptation, marginal discoloration and secondary caries.

In our intraindividual comparison of scores for RMGIC at different time intervals for secondary caries we observed that there was no significant difference in the scores at different time intervals (p > 0.05).Similar results were obtained from composite resin restorations at different time intervals (p > 0.05).

## CONCLUSION

Many options exist to repair carious primary incisors, but there is insufficient controlled, clinical data to suggest one type of restoration is superior to another. Operator preferences, esthetic demands by parents, the child’s behavior and moisture and hemorrhage control are all variables which affect the decision and ultimate outcome of whatever restorative material is chosen. Cognizance of specific strengths, weaknesses and properties of each material will enhance the clinician’s ability to make the best choice of selection for each individual situation.

Within the limits of the present *in vivo* study we conclude that:

1. Resin-modified glass ionomer cement and composite resin restorative materials showed acceptable clinical performance after 1 year in primary teeth.
2. Resin-modified glass ionomer cement and composite resin restorative materials functioned well as class III restorative materials in primary teeth.

The time period of this study was not sufficient enough to indicate the suitability of both the materials. Long-term clinical studies are required to establish the true longevity of these restorations in primary teeth.

## References

[1] (2000). US Department of Health and Human Services in America: A
report of the Surgeon General. Rockville Md: US Department of Health and Human Services.

[2] Waggoner WF, Pinkham JR (1994). Restorative dentistry for the primary dentition.. Pediatric dentistry: Infancy through adolescence..

[3] Piyapinyo S, White GE (1998). Class III cavity preparation in primary
anterior teeth: In vitro retention comparison of conventional and
modified forms. J Clin Pediatr Dent.

[4] Bowen RL (1979). Compatibility of various materials with oral tissues.
I: The components in composite restorations. J Dent Res.

[5] Craig RG (1981). Chemistry, composition and properties of composite
resins. Dent Clin North Am.

[6] Webber DL, Epstein NB, Wong JW, Tsamtsouris A (1979). A method
of restoring primary anterior teeth with the aid of a celluloid
crown form and composite resins. Pediatr Dent.

[7] de Araujo MA, Araújo RM, Marsilio AL (1998). A retrospective look
at esthetic resin composite and glass-ionomer Class III
restorations: a 2-year clinical evaluation.. Quintessence Int.

[8] Brackett MG, Dib A, Brackett WW, Estrada BE, Reyes AA (2002). One-year clinical performance of a resin-modified glass ionomer
and a resin composite restorative material in unprepared class V
restorations. Oper Dent.

[9] Ozgünaltay G, Onen A (2002). Three-year clinical evaluation of a resin
modified glass-ionomer cement and a composite resin in noncarious
class V lesions. J Oral Rehabil.

[10] Hse KM, Wei SH (1997). Clinical evaluation of compomer in primary
teeth: 1-year results. J Am Dent Assoc.

